# Genomic predictions for fillet yield and firmness in rainbow trout using reduced-density SNP panels

**DOI:** 10.1186/s12864-021-07404-9

**Published:** 2021-01-30

**Authors:** Rafet Al-Tobasei, Ali Ali, Andre L. S. Garcia, Daniela Lourenco, Tim Leeds, Mohamed Salem

**Affiliations:** 1 Computational Science Program, Middle Tennessee State University, Murfreesboro, TN 37132 USA; 2 Department of Animal and Avian Sciences, University of Maryland, College Park, MD 20742 USA; 3 Department of Animal and Dairy Science, University of Georgia, Athens, GA 30602 USA; 4 National Center for Cool and Cold Water Aquaculture, Agricultural Research Service, United States Department of Agriculture, Kearneysville, WV USA

**Keywords:** Genomic selection, GEBV, EBV, LD pruning, Predictive ability

## Abstract

**Background:**

One of the most important goals for the rainbow trout aquaculture industry is to improve fillet yield and fillet quality. Previously, we showed that a 50 K transcribed-SNP chip can be used to detect quantitative trait loci (QTL) associated with fillet yield and fillet firmness. In this study, data from 1568 fish genotyped for the 50 K transcribed-SNP chip and ~ 774 fish phenotyped for fillet yield and fillet firmness were used in a single-step genomic BLUP (ssGBLUP) model to compute the genomic estimated breeding values (GEBV). In addition, pedigree-based best linear unbiased prediction (PBLUP) was used to calculate traditional, family-based estimated breeding values (EBV).

**Results:**

The genomic predictions outperformed the traditional EBV by 35% for fillet yield and 42% for fillet firmness. The predictive ability for fillet yield and fillet firmness was 0.19–0.20 with PBLUP, and 0.27 with ssGBLUP. Additionally, reducing SNP panel densities indicated that using 500–800 SNPs in genomic predictions still provides predictive abilities higher than PBLUP.

**Conclusion:**

These results suggest that genomic evaluation is a feasible strategy to identify and select fish with superior genetic merit within rainbow trout families, even with low-density SNP panels.

**Supplementary Information:**

The online version contains supplementary material available at 10.1186/s12864-021-07404-9.

## Background

Aquaculture has great potential to enhance food security and meet the increasing consumer demand for seafood [1, 2]. However, one of the challenges is the lack of genetically improved strains of fish for aquaculture [3, 4]. Selective breeding programs can produce fish of improved genetics for heritable traits that positively impact aquaculture production [5, 6]. Breeding programs in rainbow trout have focused on the growth rate, disease resistance, and fat content [7–9].

Fillet yield has been ranked among the top traits impacting the industry returns [10]. Also, quality attributes can affect industry profitability and determine consumer’s attitudes towards the product. For instance, loss of fillet firmness contributes to fillet downgrading and economic losses to the industry [11]. Even though important for the industry, fillet yield and firmness have not received much attention because they cannot be measured directly on breeding candidates, which makes genetic selection for these traits hard to implement [12, 13]. Moderate levels of heritability estimates for fillet yield [12] and fillet firmness [13] have been reported in rainbow trout, allowing potential genetic improvement through selective breeding programs [14].

Statistical models based on phenotypes and pedigree information have been widely used in traditional genetic improvement programs to estimate EBV and identify the best selection candidates in animal populations [15]. However, applying genomic approaches has the potential to enhance and expedite genetic gains in breeding programs [16]. Although the implementation of genomic selection (GS) in livestock animals started in 2008 [17], it took more time to have this technology adopted in aquaculture species. The delayed incorporation of genomic information in rainbow trout breeding programs was mainly due to the lack of dense SNP arrays [18]. A recently developed 50 K SNP chip revealed the complex polygenic nature of fillet yield [19] and fillet firmness [13], suggesting GS as a practical strategy for rainbow trout breeding [20].

Selection based on GEBV can be performed at an early age with high accuracy, once a DNA sample for selection candidates is obtained [21]. The use of genomic information has been shown to be effective in increasing the gains in accuracy of GEBV for multiple traits in aquaculture species, including Atlantic salmon [22, 23], catfish [24], tilapia [20, 25], and rainbow trout [12, 26–29]. For rainbow trout, the accuracy of GEBV was assessed for body weight, carcass weight, fillet weight, fillet yield [12], and resistance to diseases such as bacterial cold water disease (BCWD) [26, 27], columnaris disease [28], and infectious pancreatic necrosis virus [29]. GS will allow within-family selection and hence increase the accuracy of genomic predictions and selection response, especially for lethally measured traits where within-family selection relies on traits measured in sibs of breeding candidates [25]. Additionally, GS has the potential to decrease the rates of inbreeding by selecting non-sib candidates from more families [26]. Genomic prediction can lead to higher genetic gains relative to pedigree-based selective breeding methods, which may, partially, cover the extra cost of genotyping [30]. Furthermore, GS is particularly advantageous in aquaculture species because the high fecundities of these species allow for the rapid amplification of elite genetics.

Although the use of genomic information generated from high-density SNP arrays has been demonstrated to expedite the rate of genetic gain in breeding programs, the genotyping cost is still high and alternative strategies are needed to reduce the cost of identifying elite breeding candidates [25]. Cost-effective strategies were previously assessed, yielding higher genomic prediction accuracies than those estimated using the pedigree-based PBLUP model [23, 25, 26]. The cost-reducing methods include using reduced-density SNP panels [26, 31, 32] and genotype imputation [21, 25, 33, 34]. However, imputations are prone to errors, which leads to less reliable genomic predictions [33]. Recently, the impact of low-density SNP panels on the accuracy of genomic predictions has been increasingly studied [26, 31, 32, 35, 36]. In rainbow trout, a 500 SNP panel showed higher genomic prediction accuracies for BCWD resistance (0.50–0.56) than traditional EBV (0.36) [26]. The study showed that the ssGBLUP model outperformed other genomic models, yielding high accuracy of genomic predictions with the least bias when reducing SNP panel density [26].

Recently, the accuracy of genomic prediction was assessed for fillet yield in rainbow trout using a 57 K genomic SNP panel [12]; however, the impact of reducing the SNP panel density on the gains in accuracy for fillet yield relative to the traditional PBLUP has not been investigated. Additionally, there are no reports on the benefits of using GS for quality traits such as fillet firmness in rainbow trout. Therefore, the objectives of this study were to utilize a recently developed 50 K transcribed SNP chip [19] to 1) evaluate the predictive ability of GS for fillet yield and fillet firmness in rainbow trout; and 2) investigate the impact of reducing the SNP panel density on the ability to predict fillet yield and fillet firmness.

## Results and discussion

### Phenotypes and heritability estimates

The total numbers of phenotyped fish for fillet yield and shear force were 775 and 772, respectively, and varied per year-class (YC) with 471 fish in YC 2012, and 304 fish in YC 2010. Descriptive statistics of the data are provided in Table 1. A slightly higher fillet yield and shear force were observed for fish from the YC 2012 compared to that from YC 2010. A small phenotypic correlation (R = 0.14) was observed between fillet yield and shear force in this study.

**Table 1:** Descriptive statistics and the number of genotyped and phenotyped fish per year class

Year class	Fillet yield (%)		Shear force (g/g)	
	2010	2012	2010	2012
Mean	48.21	49.59	448.92	491.91
Median	48.45	49.68	450.91	487.01
Max	55.30	55.62	724.39	822.67
Min	34.77	39.22	166.98	234.24
SD	2.64	1.94	87.53	81.44
CV%	5.48	3.91	5.13	6.04
Phenotyped	304	471	301	471
Genotyped	400	558	400	558

A total of 1572 genotyped fish passed the quality check (QC) and were available for this study (four samples were removed due to fish ID conflicts). The total number of genotyped individuals from YC 2010 and 2012 were 400 and 558, respectively (Table 1). In addition, 380 fish were genotyped before YC 2010, whereas 234 were genotyped after YC 2012. No phenotypic data were available for fish produced before YC 2010 or after YC 2012 (Table 1). All fish had complete pedigree information dating back to YC 2002. Fig. S1 depicts a heatmap of the pedigree (A22) and the genomic (G) relationship matrices for data generated from the population used in this study.

In this study, the heritability estimates were calculated based on all data from YC 2010 and YC 2012 using PBLUP and ssGBLUP models and are shown in Table 2. Variance components are provided in Additional file 1 (Table S1). The estimated heritability values using both methods were higher for fillet firmness than for fillet yield (Table 2). Other studies reported a slightly higher heritability (0.34–0.36) of fillet yield in other rainbow trout populations [12, 37]. The estimated heritability values indicate a moderate additive genetic component for these traits. Garcia-Ruiz et al. (2016) showed that genomic information can help to increase accuracy even for lowly heritable traits. Additionally, increased accuracies in a trait with h^2^ = 0.14 was reported in Atlantic salmon using GBLUP [22], which provides evidence for the feasibility of GS in the current NCCCWA fish population.

**Table 2:** Heritability, predictive ability, and regression coefficient of adjusted phenotype on (G) EBV for fillet yield and fillet firmness using PBLUP and ssGBLUP

	Fillet yield		Fillet firmness	
	PBLUP (SE)	ssGBLUP (SE)	PBLUP (SE)	ssGBLUP (SE)
Heritability	0.25 (0.073)	0.26 (0.073)	0.38 (0.082)	0.38 (0.082)
Predictive ability	0.20 (0.078)	0.27 (0.077)	0.19 (0.079)	0.27 (0.077)
Regression coefficient (b1)	0.96	0.97	0.79	0.88

### Genomic predictions using 50 K SNP panel

The predictive ability of the genomic model was evaluated using a five-fold cross-validation scheme. Using five replicates helps to minimize errors that could be generated due to a single-fold sampling [28]. Table 2 shows the average predictive ability for fillet yield and firmness under the five-fold cross-validation strategy. For both traits, using genomic information through ssGBLUP enhanced the ability to predict fish performance relative to PBLUP. Genomic information has a greater impact on the predictive ability of lethally-measured traits than traits that can be directly measured on fish [12, 24]. In this study, genomic information increased predictive ability by 35 and 42%, compared to PBLUP, for fillet yield and firmness, respectively. Gonzalez-Pena et al. [12] also reported higher accuracy of GEBV compared to EBV; 0.55 and 0.13, respectively. Similar gains through GS have been achieved in other aquaculture species. For instance, a 29% increase in predictive ability, relative to PBLUP, was reported for residual carcass weight in channel catfish [24].

Variable gains of accuracy using genomic information were reported in Atlantic salmon for lice resistance (52%), fillet color (22%) [22], and growth traits (20%) [23]. The considerable variation in the relative improvement of accuracy between the traits is consistent with the difference in predictive ability between fillet yield and firmness in the current study. Even though high predictive abilities have been achieved by applying genomic information in this study, using a higher number of fish in the training population would help to achieve higher prediction accuracies. Low predictive abilities (0.46–0.49) for BCWD phenotypes in trout were observed using ssGBLUP compared to PBLUP (0.50) when a small training sample size (*n* = 583) was used [38]. Incorporating more genotyped and phenotyped fish in the analysis improved the accuracy of the GEBVs by ~ 80% [27].

The regression coefficients (b1), representing inflation for fillet yield and firmness, are shown in Table 2. For fillet firmness, GEBV was less inflated than EBV. These results are consistent with results reported for harvest weight and residual carcass weight in catfish [24] and BCWD resistance in rainbow trout [27], showing that the genomic information provides not only more accurate but also less biased evaluations. In this study, variance components estimated from the whole dataset were used. Using the same variance components for a five-fold cross-validation strategy yielded less inflated GEBV for fillet yield, close to 1 (b1 = 0.97), than that of the harvest weight (b1 = 0.92) and residual carcass weight (b1 = 0.91) in catfish [24], and BCWD survival status in rainbow trout (b1 = 0.86) [27]. On the other hand, breeding values for fillet firmness were more inflated (b1 = 0.88) than those for fillet yield. Updating the variance components for the training datasets in catfish have been suggested to reduce the inflation of the genomic evaluations [24].

Fish used in this study were sampled from a genetic line selected for a high growth rate. Previously, when fillet yield and shear force phenotypes were regressed on body weight, coefficient of determination (*R*^2^) values of 0.56 and 0.01 were observed, respectively [39]. Selection for growth in this population might have less representation of fish with low-ranked phenotype for fillet yield. Fish with phenotypes used in the study were sampled in a manner that captures between- and within-family variation for growth performance although. In an admixed population of Atlantic salmon, fish families produced from selection lines showing extreme phenotypes for lice resistance were over-represented among the genotyped fish leading to inflation in the between-family variation and less reliability of the GS model [22].

### Linkage disequilibrium and effective population size

**Fig. 1:**
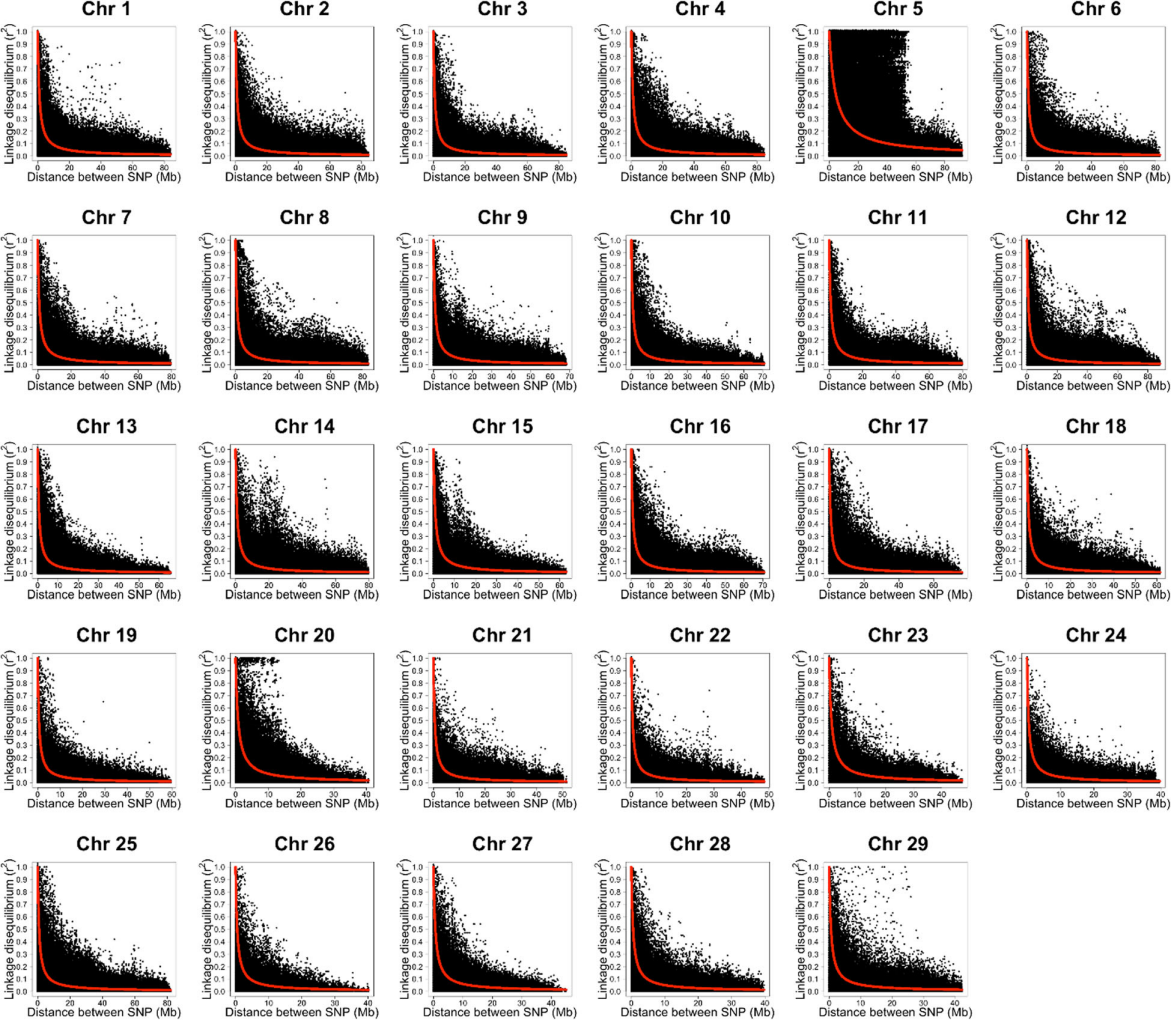
Plot of LD decay estimated for all chromosomes using 50 K SNP panel in rainbow trout from the NCCCWA genetic line. Chromosome 5 showed a long-range LD extended over 20 Mb

Long-range linkage disequilibrium (LD) and small effective population size (*Ne*) provided evidence of the possibility to reduce the marker density needed for GS in catfish and rainbow trout selectively-bred for BCWD resistance [24, 26]. When the historical LD is weak, the accuracy of genomic prediction decreases [40]. In addition, higher genomic prediction accuracies are associated with small *Ne* [24, 41, 42]*.* LD and *Ne* were estimated for the rainbow trout population used in this study (Table 3). The mean LD per chromosome ranged from 0.21 to 0.34, with an overall genome-wide average *r*^2^ of 0.26. Twenty-two chromosomes showed a mean *r*^2^ ≥ 0.25. The LD (*r*^2^ = 0.26) in the current fish population was consistent with LD (*r*^2^ = 0.27) reported in a Troutlodge, Inc., May-spawning, odd-year line [26]. Chromosomes 5 and 23 had the highest mean LD estimate (*r*^2^ = 0.34) followed by chromosome 1 (*r*^2^ = 0.32). Conversely, chromosomes 6 (*r*^2^ = 0.21) and 19 (*r*^2^ = 0.22) had the lowest mean LD estimates. LD decay with distance was estimated for all the rainbow trout chromosomes using only high-quality anchored SNPs (Fig. 1). The LD decay plots show a long-range LD in all chromosomes. Interestingly, the strongest LD appeared on chromosome 5 and extended over 20 Mb. In agreement with our results, Vallejo et al. [26] identified long-range LD spanning over 1 Mb in all chromosomes and over 25 Mb on chromosome 5 in a commercial rainbow trout population. This strong LD on chromosome 5 was likely due to a large chromosomal double inversion, which prevents recombination in heterozygous fish [26, 43]. Also, recent admixture events [22] and recombination interference on the male chromosome were shown to contribute to long-range LD in rainbow trout and other salmonids [12]. The population structure analysis suggested nine genetically different groups in the current population (data not shown), thus supporting population admixture as a contributor to the long-range LD observed in this study. In contrast, a Tasmanian Atlantic salmon population that was originated from a single founder strain showed short-range LD [44].

**Table 3:** Linkage disequilibrium and effective population size by chromosome. Chromosome 5 had the highest mean LD estimate (*r*^2^) and the smallest effective population size

CHR	Number of SNPs	Size (bp)	Mean (*r*^2^)	*Ne*
1	1298	80,480,703	0.32	81
2	1580	85,221,383	0.26	92
3	1409	84,915,088	0.25	104
4	1380	84,297,858	0.27	80
5	2060	91,890,047	0.34	18
6	1189	77,071,016	0.21	105
7	1278	79,714,361	0.24	91
8	1655	83,683,676	0.27	94
9	1339	68,350,947	0.30	110
10	1169	64,390,894	0.26	114
11	1119	79,489,160	0.26	87
12	1711	81,222,362	0.24	98
13	1069	47,531,856	0.25	128
14	1284	80,268,421	0.26	94
15	913	59,307,523	0.25	100
16	1572	70,790,504	0.27	113
17	1379	76,497,411	0.26	104
18	1133	61,193,526	0.25	125
19	872	58,039,566	0.22	147
20	677	40,981,438	0.26	105
21	783	51,606,644	0.23	165
22	792	48,370,041	0.25	180
23	602	48,805,734	0.34	97
24	633	40,198,439	0.25	171
25	1330	82,450,733	0.26	97
26	453	30,795,589	0.23	166
27	657	45,210,144	0.27	127
28	843	40,702,581	0.23	153
29	731	42,409,034	0.26	141
Average	1135	65,030,575	0.26	113
SD	389	17,941,345	0.031	34

The mean estimated effective population size based on LD was *Ne* = 113 (Table 3). An average *Ne* of 145 was reported in the early generations of the NCCCWA rainbow trout selective breeding program and was expected to decline with selection [45]. The estimated *Ne* in this population is comparable to that estimated in other livestock species; Jersey cattle (*Ne* = 101), Angus cattle (*Ne* = 113), Holstein cattle (*Ne* = 149) [46] and a commercial rainbow trout population (*Ne* = 155) [26], and it is considerably larger than the *Ne* estimated for catfish (*Ne* = 27) [24], swine (*Ne* = 32), and chicken (*Ne* = 44) [46]. Chromosome 22 had the largest effective population size (*Ne* = 180) followed by chromosome 24 (*Ne* = 171). Chromosomes 5 (*Ne* = 18) and 4 (*Ne* = 80) had the smallest effective population size. In the early generations produced at NCCCWA, an *Ne* range of 75.51–203.35 was reported, and the authors suggested the presence of suites of genes on each chromosome that are disproportionately under selection pressure [45]. The *Ne* in this population may have limited the predictive ability because only a small number of genotyped fish were available for computing genomic predictions. Based on the average effective population size (*Ne* = 113) and trout genome length (*L* = 32.3 M), the number of independent chromosome segments (*Me*) for this population was ~ 14,600, according to the equation *Me* = 4*NeL* [47]. In a commercial rainbow trout population, an *Me* = 20,026 was reported [26]. Overall, our results suggest the feasibility of reducing the SNP panel size, which can reduce the genotyping cost. In the next section, further investigation was conducted to determine the impact of reducing the SNP panel size on the predictive ability of GEBV in the current rainbow trout population.

### Impact of reducing the SNP panel density

There is an interest from the aquaculture industry to implement GS in breeding programs. However, genome-wide genotyping using high-density SNP chips may be cost-prohibitive, especially for small and mid-size hatcheries and companies; therefore, a cost-efficient genotyping is necessary.

In the current study, two approaches have been adopted to evaluate the effect of reducing the density of the SNP panel on the genomic predictive ability for fillet yield and firmness.

#### Reducing the SNP panel density based on LD

In the first approach, LD-based SNP pruning was used to reduce the density of SNP panel throughout the genome [48]. Five LD values were used to prune SNPs (*r*^2^ > 0.7, > 0.4, > 0.1, > 0.05, and > 0.01). The predictive ability for fillet yield, based on LD, was reasonably well maintained with the reduction of the SNP panel density down to ~ 11 K SNPs (Table 4). However, further reduction of the marker density, below 11 K SNPs, led to lower predictive abilities than those estimated using the PBLUP model (0.20). Similar results have been reported in other aquaculture populations/species. For instance, a moderate decrease in genomic accuracy was reported when reducing the SNP density down to 10 K SNPs in a trout population selected for BCWD resistance [26]. Also, increasing SNP density above ~ 10 K SNPs in Atlantic salmon showed no improvement in genomic accuracy for growth traits [23]. This can be related to the theory of dimensionality of genomic information, where the number of animals and SNP needed to achieve a reasonable level of accuracy approaches *4NeL* [49, 50], which was 14,600 in our study.

**Table 4:** Predictive ability and regression coefficient of adjusted phenotype on (G) EBV for fillet yield and firmness with reduced-SNP panel density based on LD

LD (*r*^2^)	N of SNPs	Predictive ability ± SE		Regression coefficient (b1)	
		GEBV		GEBV	
		Fillet yield	Fillet firmness	Fillet yield	Fillet firmness
50 K chip	35,303	0.27 ± 0.077	0.27 ± 0.077	0.97	0.88
> 0.7	20,042	0.27 ± 0.077	0.27 ± 0.077	0.99	0.90
> 0.4	11,433	0.26 ± 0.077	0.26 ± 0.078	0.98	0.88
> 0.1	1153	0.19 ± 0.079	0.27 ± 0.077	0.71	0.79
> 0.05	497	0.17 ± 0.079	0.24 ± 0.078	0.57	0.67
> 0.01	128	0.07 ± 0.080^a^	0.02 ± 0.080^a^	0.21	0.04

^a^indicates a significant difference between the low-density SNP panel and the 50 K SNP panel in predicting the future performance of fish (t-test *p*-value < 0.05)

In the case of fillet firmness, a reduction of the SNP density down to 1153 K SNPs yielded the same predictive ability (0.27) as the full 50 K SNP panel; however, GEBV were more inflated. Interestingly, further reduction of the SNP density down to 497 SNPs yielded predictive abilities (0.24) that, although were lower than the 50 K panel, still outperformed the traditional PBLUP model (0.19) (Table 4). Recently, gains of accuracy relative to traditional PBLUP, using 500 SNP panel, were reported for BCWD in rainbow trout. Such gains in accuracy with a significantly reduced SNP panel was attributed to the long-range LD in the studied population [26].

#### Reducing SNP density based on the percentage of additive genetic variance explained by SNPs

The second approach involved reducing the density of the panel based on the percentage of additive genetic variance explained by SNPs for each trait, which were already determined in our previous publications using weighted single-step genomic best linear unbiased prediction (wssGBLUP) analysis [13, 19]. Reduced SNP panels with the percentage of additive genetic variance between > 0.05% and > 1.8% were used to evaluate the predictive ability using the five-fold cross-validation strategy. Tables 5 and 6 show the predictive ability for fillet yield and firmness using those reduced SNP panels. For fillet yield, reduction of the SNP marker density down to ~ 9 K SNPs yielded the same predictive ability (0.27) that was obtained using the 50 K panel. Vallejo et al. [26] reported that SNP panel densities less than 20 K SNPs are suitable for GS in rainbow trout. Simulation studies in plants and livestock showed that marker densities higher than ~ 10 K SNPs had little or no improvement in genomic accuracy [51, 52]. Interestingly, further reduction of the number of SNPs down to ~ 800 SNPs caused a minimal decrease in the predictive ability for fillet yield (0.26). For fillet firmness, reducing the number of SNPs down to 10 K SNPs had a small increase in predictive ability (0.30) compared to the 50 K SNP panel (0.27). Interestingly, the inflation of GEBV greatly improved for firmness without a significant change in fillet yield. For both traits, the predictive ability of GEBV for all SNP panels down to ~ 800 SNPs was higher than those estimated using the pedigree-based model. Overall, prioritizing SNPs based on their percentage of additive genetic variance explained allowed a high reduction of the SNP density, down to ~ 800 SNPs, while maintaining the potential to enhance the accuracy of genomic predictions in this population, but with a trade-off of increasing inflation.

**Table 5:** Predictive ability and regression coefficient of adjusted phenotype on (G) EBV for fillet yield with reduced-SNP panel density based on the proportion of additive genetic variance explained by SNPs

% Additive Genetic Variance explained by SNPs	N of SNPs	Predictive ability ± SE	Regression coefficient (b1)
		GEBV	GEBV
50 K	35,303	0.27 ± 0.077	0.97
> 0.05	16,381	0.25 ± 0.078	0.87
> 0.1	9493	0.27 ± 0.077	0.96
> 0.5	857	0.26 ± 0.077	0.85
> 1.0	232	0.19 ± 0.079	0.59
> 1.5	102	0.10 ± 0.080^a^	0.34
> 1.8	68	0.12 ± 0.080^a^	0.44

^a^indicates a significant difference between the low-density SNP panel and the 50 K SNP panel in predicting the future performance of fish (t-test *p-*value < 0.05)

**Table 6:** Predictive ability and regression coefficient of adjusted phenotype on (G) EBV for fillet firmness with reduced-SNP panel density based on the proportion of additive genetic variance explained by SNPs

% Additive Genetic Variance explained	N of SNPs	Predictive ability ± SE	Regression coefficient (b1)
		GEBV	GEBV
50 K	35,303	0.27 ± 0.077	0.88
> 0.05	17,592	0.27 ± 0.077	0.91
> 0.1	10,533	0.30 ± 0.077	0.99
> 0.5	808	0.23 ± 0.078	0.70
> 0.8	273	0.17 ± 0.079	0.47
> 1.0	139	0.12 ± 0.080^a^	0.33

^a^indicates a significant difference between the low-density SNP panel and the 50 K SNP panel in predicting the future performance of fish (t-test *p*-value < 0.05)

Using the proportion of additive genetic variance explained by SNPs for pruning was more effective than the LD approach for fillet yield. Conversely, the LD approach for SNP pruning was more effective in predicting fillet firmness with low-density SNP panels. This could be attributed to the different genetic architecture between traits combined with the number of SNPs retained for analysis.

Altogether, reducing the SNP density to about 10,000 SNPs would likely help to reduce the genotyping cost of implementing GS in this population with no loss in predictive ability and no bias. A further reduction to around 1000 SNP would have a small impact on predictive ability, but GEBV may have an increased level of bias depending on which method was used to reduce SNP density.

## Conclusions

This study reveals the impact of using genomic information on progressing the rainbow trout breeding programs for fillet yield and firmness. Using genomic information improves the ability to predict future performance and reduces the inflation of breeding values. The long-range LD detected in this study, partially due to population admixture, is likely contributing to the high genomic predictive ability in case of the reduced density SNP panels. Reducing the SNP panel density to approximately 10,000 SNPs is a feasible strategy to help to reduce the cost of implementing GS in rainbow trout.

## Methods

### Fish population and phenotypes

The fish population and sample collection were previously described in detail [53]. The selective breeding program has been established in 2004 and underwent five generations of selection for body weight. Details on how the population was formed and selected for growth can be found in [8, 54]. Phenotypic records on fillet yield and shear force were collected from the third and fourth generations, comprising YC 2010 and 2012. A total of 775 fish representing 76 full-sib families from YC 2010 and 98 families from YC 2012, were used. To maintain unique pedigree information, each family was kept in a separate 200-L tank until they were tagged at ~ 5-month post-hatching. Subsequently, fish were reared together in 800-L tanks and fed a commercial fishmeal-based diet.

For phenotyping, fish were harvested over five consecutive weeks (one fish/family/week), yielding 5 harvest groups. The YC 2010 fish were 410 to 437 days old at harvest with a mean body weight of 985 ± 239 (g). Fish from the YC 2012 were 446 to 481 days old, with a mean body weight of 1803 ± 305 (g). Head-on gutted carcasses were manually processed into skinless fillet. The trimmed fillet was weighed, and fillet yield was calculated as a percent of whole-body weight. The fish (YC 2010 & YC 2012) had an average fillet yield of 48.91 ± 2.42 (%). The shear force of a cooked fillet section was used to measure the fillet firmness as fully described in a previous publication [55].

### Genotyping data and quality control check

In the current study, we used a 50 K gene-transcribed SNP chip that has recently been developed for rainbow trout [19]. A total of 1728 fish were genotyped as previously described [13, 19]. Affymetrix SNPolisher software was used at the default parameters to perform quality control and filter out samples that did not meet the threshold filtration criteria. In addition, about 5 K SNPs that were not anchored to the newly assembled rainbow trout genome were filtered out because LD calculation (explained in the next sections) requires physically mapped SNPs. Anchored SNPs were subjected to QC analysis using PREGSF90 software, which belongs to the BLUPF90 family [56]. After QC, 35,303 SNPs (70.6%) were retained, those SNPs had minor allele frequency (MAF) > 0.05, call rate for SNP > 0.90, and deviation from the Hardy–Weinberg equilibrium (HWE) < 0.15. The filtered SNPs were used both for GS and LD analysis.

### Model and analysis

Two single-trait mixed models were used for fillet yield and shear force (fillet firmness) as follows:

$$ \mathbf{y}=\mathbf{Xb}+{\mathbf{Z}}_1\mathbf{a}+{\mathbf{Z}}_2\mathbf{c}+\mathbf{e}. $$

Where **y** is a vector of phenotypes (fillet yield or fillet firmness), **b** is a vector of fixed effects of hatch-year and harvest group, **a** is a vector of additive direct genetic effect, **c** is a vector of random of common environmental effect (i.e., family effect), and **e** is the vector of residuals. Incidence matrices for effect contained in **b**, **a**, and **c** are represented by **X**, **Z**_1_, and **Z**_2_, respectively.

The BLUPF90 from the BLUPF90 family of programs was used to perform both traditional PBLUP and ssGBLUP analyses [56]. The ssGBLUP model uses both pedigree and genomic information to calculate GEBV. Those two sources of information are combined into a realized relationship matrix (**H**), where the inverse (**H**^−1^) replaces the inverse of the pedigree relationship matrix (**A**^−1^) in the BLUP mixed model equations. The **H**^−1^ was described in [57] as follows:

$$ {\mathbf{H}}^{-1}={\mathbf{A}}^{-1}+\left[\begin{array}{cc}0& 0\\ {}0& {\mathbf{G}}^{-1}-{\mathbf{A}}_{22}^{-1}\end{array}\right] $$

Where ${\mathbf{A}}_{22}^{-1}$ is the inverse of the pedigree relationship matrix for genotyped animals.

**G**^−1^ is the inverse of the genomic relationship matrix, constructed as we previously described [24].

$$ \mathrm{G}=\frac{\mathrm{MDM}^{\prime }}{2\sum {\mathrm{p}}_{\mathrm{j}}\left(1-{\mathrm{p}}_{\mathrm{j}}\right)^{\prime }} $$

Where M is a matrix of genotypes centered by twice the current allele frequencies (p); j is the jth locus; D is a diagonal matrix of SNP weights with a dimension equal to the number of SNPs. All SNPs were assigned to have homogeneous weights, i.e., D was an identity matrix (I).

Variance components were estimated using single-trait models with all data, with and without genomic information for both traits, using AIREMLF90 [58]. However, pedigree-based variance components, estimated with all the data, were used in the validation study to have fair comparisons between PBLUP and ssGBLUP. Heritabilities were calculated as:

$$ {h}^2=\frac{\sigma_a^2}{\sigma_a^2+{\sigma}_c^2+{\sigma}_e^2} $$

where ${\sigma}_a^2$ is the additive genetic variance, ${\sigma}_c^2$ is the common environmental variance, and ${\sigma}_e^2$ is the residual variance.

### Cross-validation

To evaluate predictive abilities of both traditional pedigree-based and genomic evaluations, as well as the impact of different SNP densities, five-fold cross-validation was conducted [24, 28, 59]. The genotyped animals with phenotypes (fillet yield *N* = 775; shear force *N* = 772) were randomly split into five mutually exclusive folds/groups. Then, phenotypes were removed from the data, one group at a time from the validation groups (fillet yield *N* = 155; shear force N = ~ 154). The remaining animals (i.e., training group) were used to predict the future performance of the validation group. Results were presented as the overall mean of the 5 replicates. This cross-validation approach was chosen given the small number of genotyped animals with phenotypes.

To calculate the predictive ability, phenotypes were adjusted for fixed effects (y*) as we previously described in [54]. Predictive ability was defined as the Pearson’s correlation between adjusted phenotypes (y*) and (G)EBV.

Predictive ability = *cor* [(G) EBV, y^∗^].

Further, inflation was assessed as the regression coefficient (b1) of adjusted phenotypes (y*) on (G) EBV as follows:

y^∗^ = b0 + b1 × (G) EBV + e.

where b1 < 1 indicates inflation and b1 > 1 indicates deflation of (G)EBV [24].

### Linkage disequilibrium and effective population size

The LD was calculated using PREGSF90 [56] according to the following equation:

$$ {r}^2=\frac{{\mathrm{D}}^2}{P_{\mathrm{A}}{P}_{\mathrm{a}}{P}_{\mathrm{B}}{P}_{\mathrm{b}}} $$

where *P*_A,_ *P*_a,_ *P*_B, and_*P*_b_ represent the allele frequencies; *D* = *P*_AB_ − *P*_A_*P*_B_ where *P*_AB_ is the frequency of the genotype AB. The mean LD was estimated for each chromosome as the average estimate of *r*^*2*^ from all pairwise SNPs [26].

For each chromosome, LD decay with the distance between SNP markers was calculated by fitting Sved’s equation [60] as follows:

$$ E\left[{r}_t^2\right]=\frac{1}{\left(1+4{N}_{et}{d}_{ij}\right)} $$

where *d*_*ij*_ is the distance between the SNP-marker pair *i* and *j*; *N*_*et*_ is the effective population size for chromosome t; *N*_*e*_ was calculated using the equation proposed by Saura et al. [61]:

$$ {N}_{et}={\left.\left(4\right.{\mathrm{d}}_t\right)}^{-1}\ \left[{\left({r}_t^2-{\mathrm{n}}^{-1}\right)}^{-1}-\upalpha \right] $$

where *d*_*t*_ is the average length of chromosome t in Morgan, *r*^2^ is the average LD of chromosome t, (n)^−1^ represents the adjustment term for the sample size, and α is a fixed parameter that equals to 1 in the absence of mutations or to 2 in the presence of mutations. In this study, α = 2 was used.

### Reducing SNP density based on LD

PLINK 1.9 [48] was used to generate reduced/pruned SNP subsets based on variable LD values of 0.7, 0.4, 0.1, 0.05, and 0.01. SNP pruning was achieved using the command line (plink –file data –indep-pairwise 50 5 *r*^2^). In brief, LD was calculated between each pair of SNPs within a genomic window of 50 SNPs. A pair of SNPs was removed if the LD between the two pairs of SNPs exceeded the LD value, e.g., 0.7. The window was shifted 5 SNPs forward, and the procedure was repeated. The command line was re-executed using the four other *r*^2^ values. Each one of the five resulting pruned SNP subsets with 20,042, 11,433, 1153, 497, and 128 SNPs were used in genomic predictions.

### Reducing SNP density based on the percentage of additive genetic variance

The second approach used to reduce the density of the SNP panel was based on the percentage of additive genetic variance explained by SNPs in each trait. The SNP variances were previously computed using wssGBLUP for fillet yield and fillet firmness [13, 19]. In this approach, SNPs were clustered into five to six groups based on the percentage of additive variance explained, with clusters ranging from > 0.05% to > 1.0%. Accordingly, the reduced number of SNPs of each group was used to evaluate the predictive ability using the five-fold cross-validation strategy.

## Supplementary Information

**Additional file 1: Figure S1:** A heat map for Pedigree matrix (A22) and Genomic matrix (G) where the color density reflects the relationship between individuals used in this study. **Table S1:** Genetic parameters of fillet yield and fillet firmness using ssGBLUP.

## Data Availability

All datasets generated for this study are included in the manuscript and/or the additional file. The genotypes (ped and map files) and phenotypes are available in our previous publication [13].

## Abbreviations

QTL: Quantitative Trait Loci
GEBV: Genomic Estimated Breeding Values
PBLUP: Pedigree-based Best Linear Unbiased Prediction
EBV: Estimated Breeding Values
ssGBLUP: Single-step Genomic Best Linear Unbiased Prediction
GS: Genomic Selection
BCWD: Bacterial Cold Water Disease
YC: Year-Class
QC: Quality Check
NCCCWA: National Center of Cool and Cold Water Aquaculture
LD: Linkage Disequilibrium
HWE: Hardy–Weinberg Equilibrium
wssGBLUP: Weighted single-step Genomic Best Linear Unbiased Prediction
