# Supplementary material for: Genomic predictions for fillet yield and firmness in rainbow trout using reduced-density SNP panels
Source: BMC Genomics. 2021 Jan 30;22:92. doi: 10.1186/s12864-021-07404-9 (PMC7847601; doi:10.1186/s12864-021-07404-9)
Supplement: Supplementary file 1 — Additional file 1: Figure S1: A heat map for Pedigree matrix (A22) and Genomic matrix (G) where the color density reflects the relationship between individuals used in this study. Table S1: Genetic parameters of fillet yield and fillet firmness using ssGBLUP. [file 12864_2021_7404_MOESM1_ESM.docx]

**GENOMIC PREDICTIONS FOR FILLET YIELD AND FIRMNESS IN RAINBOW TROUT USING REDUCED-DENSITY SNP PANELS**

Rafet Al-Tobasei^1^, Ali Ali^2^, Andre L. S. Garcia^3^, Daniela Lourenco^3^, [Tim](https://www.ncbi.nlm.nih.gov/pubmed/?term=Wiens%20GD%5BAuthor%5D&cauthor=true&cauthor_uid=25620978) Leeds^4^, and Mohamed Salem^2^

^1^Computational Science Program, Middle Tennessee State University, Murfreesboro, TN 37132, U.S.

^2^Department of Animal and Avian Sciences, University of Maryland, College Park, Maryland 20742 U.S.

^3^Department of Animal and Dairy Science, University of Georgia, Athens, GA 30602, U.S.

^4^National Center for Cool and Cold Water Aquaculture, Agricultural Research Service, United States Department of Agriculture Kearneysville, WV, U.S.

Corresponding author: Mohamed Salem

E-mail: mosalem@umd.edu


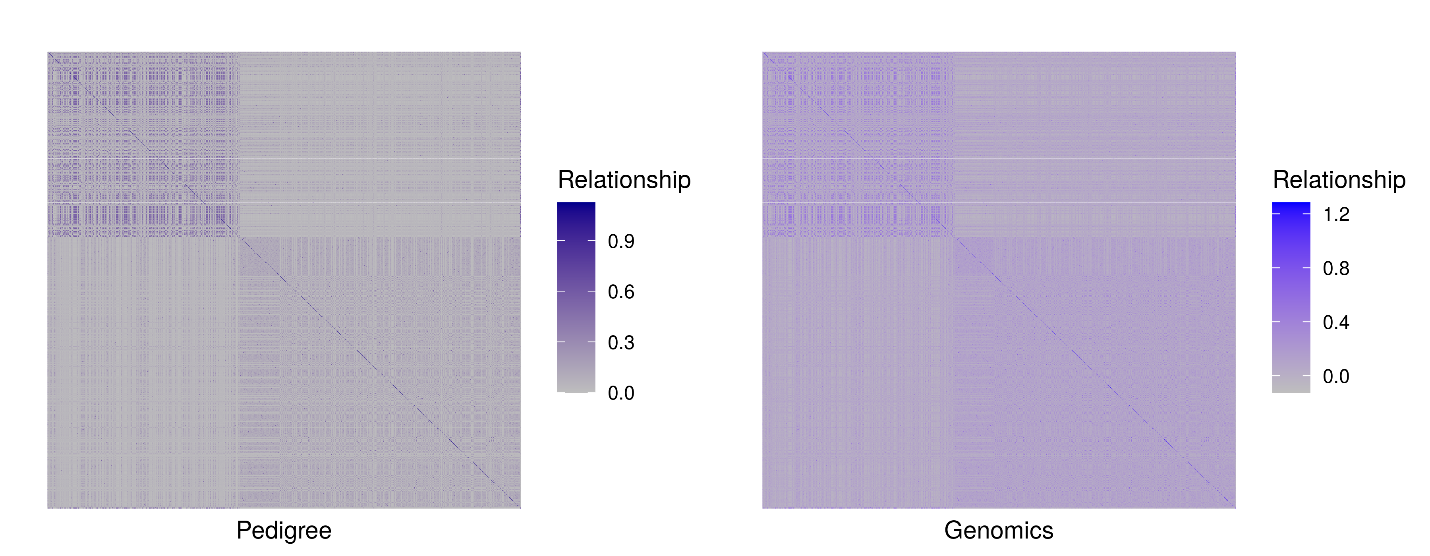


Figure S1: A heat map for Pedigree matrix (A22) and Genomic matrix (G) where the color density reflects the relationship between individuals used in this study.

Table S1: Genetic parameters of fillet yield and fillet firmness using ssGBLUP.

| Phenotype | σ^2^_a_ (SE) | σ^2^_c_ (SE) | σ^2^_e_ (SE) | h^2^ (SE) |
| --- | --- | --- | --- | --- |
| Fillet yield | 1.22 (0.361) | 0.61 (0.192) | 2.67 (0.228) | 0.27 (0.073) |
| Fillet firmness | 2579.40 (653.13) | 84.25 (222.73) | 4049 (380.51) | 0.38 (0.082) |
